# Two sources of bias explain errors in facial age estimation

**DOI:** 10.1098/rsos.180841

**Published:** 2018-10-17

**Authors:** Colin W. G. Clifford, Tamara L. Watson, David White

**Affiliations:** 1 School of Psychology, UNSW Sydney, Sydney, New South Wales 2052, Australia; 2 School of Social Sciences and Psychology, Western Sydney University, Sydney, New South Wales, Australia

**Keywords:** social vision, person perception, ageing, face perception, serial dependency

## Abstract

Accurate age estimates underpin our everyday social interactions, the provision of age-restricted services and police investigations. Previous work suggests that these judgements are error-prone, but the processes giving rise to these errors are not understood. Here, we present the first systematic test of bias in age estimation using a large database of standardized passport images of heterogeneous ages (*n* = 3948). In three experiments, we tested a range of perceiver age groups (*n* = 84), and found average age estimation error to be approximately 8 years. We show that this error can be attributed to two separable sources of bias. First, and accounting for the vast majority of variance, our results show an assimilative serial dependency whereby estimates are systematically biased towards the age of the preceding face. Second, younger faces are generally perceived to be older than they are, and older faces to be younger. In combination, these biases account for around 95% of variance in age estimates. We conclude that perception of age is modulated by representations that encode both a viewer's recent and normative exposure to faces. The finding that age perception is subject to strong top-down influences based on our immediate experience has implications for our understanding of perceptual processes involved in face perception, and for improving accuracy of age estimation in important real-world tasks.

## 1. Introduction

Faces are uniquely social stimuli, carrying visual information that is critical to our normal social functioning. As a result, a substantial amount of research has examined mechanisms responsible for accurate perception of facial identity and expression [1]. Substantially less attention has been paid to the perception of facial age, despite the profound social significance of these judgements. Perceived age is critical in defining social structures across cultures, in distinguishing between group members and forming stereotypes [2,3]. Perceived age of a face has a substantial impact on the social perceptions we form of people, affecting, for example, perceptions of warmth and dominance [4], which account for a substantial proportion of variance in the first impressions we form of unfamiliar faces [5]. Indeed, a recent study estimated perceived facial age to account for around 20% of variance in social evaluations of faces [6].

Given the universal social importance of age estimation one might expect that perceptual systems would be well attuned to this source of facial information. Early work examining age estimation from faces noted that people make reliable estimates, with error in the range of 3–4 years [7–9]. However, these studies were based on very small data samples, both in terms of faces used in age estimation tasks and the participants that performed them. In more recent tests using larger samples of faces, and participants of more heterogeneous age, it has become clear that perceptual estimates of age are more prone to error than originally thought, with mean error around 6 years [10,11]. One study also shows poor performance outside of the laboratory, with shopkeepers misjudging 38% of 16-year-old boys and 56% of 16-year-old girls to be of legal drinking age, i.e. at least 18 [12]. Errors of this magnitude can have a substantial impact on other critical tasks. In child exploitation investigations, for example, it is often necessary to estimate facial age of victims by reviewing large volumes of image data [13]. Developing an understanding of why these errors in age judgements arise is necessary to improve the reliability of these important decisions.

Recent studies suggest that biases arising from perceptual experience contribute to errors observed in age estimation. For example, estimates of facial age appear to be biased towards the middle-aged faces, resulting in younger faces appearing older than they are and older faces appearing younger [14]. This result was particularly clear when images were viewed through visual noise, suggesting that in conditions of uncertainty, perception defaults to stored representations that reflect the normative state of our experience. This account is also consistent with the finding that people tend to be more accurate when estimating the age of people similar in age to themselves [8,10,11]. Because we tend to have more extensive experience with faces of our own-age group, normative representations are more supportive of perceptual processing when faces are from our own-age group.

In a separate line of investigation, perceptual biases have been shown to arise over shorter time frames, based on stimuli experienced immediately prior to the current stimulus. Serial dependencies can be both ‘assimilative’, whereby the current stimulus is biased to appear more similar to the previous stimulus, or ‘contrastive’, whereby perception is biased away from the previous stimulus [15]. Although these effects have not yet been observed in age estimation tasks, they have been shown to bias both facial identity [16], gender [17], expression [18] and attractiveness judgements [18–22]. This raises the possibility that serial dependencies interact with normative representations to produce biases in the perception of facial age.

Here, we address the separate contributions of these sources of bias. We present the first systematic examination of biases in face age estimation using a large database of passport images of heterogeneous age. In three separate experiments, we asked young adult and older adult participants to provide numerical age estimates from passport photographs. These photographs were of 3968 Australian citizens who had agreed, as part of the passport application process, to have their photograph used in academic research. This dataset provided a naturalistic resource for examining human age judgements, and enabled us to examine the accuracy of age estimates to faces across a uniform distribution of ages from 7 to 70. To investigate the effect of uncertainty on biases in age perception, images were presented at one of three levels of masking.

In previous work, an important obstacle to estimating the accuracy of age across the lifespan has been the absence of large and diverse image sets. Given that people age at different rates, using small sets of faces means that accuracy of age estimation at any given age could be esoteric to the specific identities pictured. For example, one person may have made particularly poor lifestyle choices or have a particular genetic disposition towards skin ageing [23], leading to their appearance being atypical for their age and therefore inaccurate age estimates. Some studies have overcome this by using generative computer models that produce images varying on an age parameter [14,24]. However, this approach constrains the generality of findings, raising the possibility that effects are specific to a particular generative model or the faces used to create the model.

**Figure 1.**
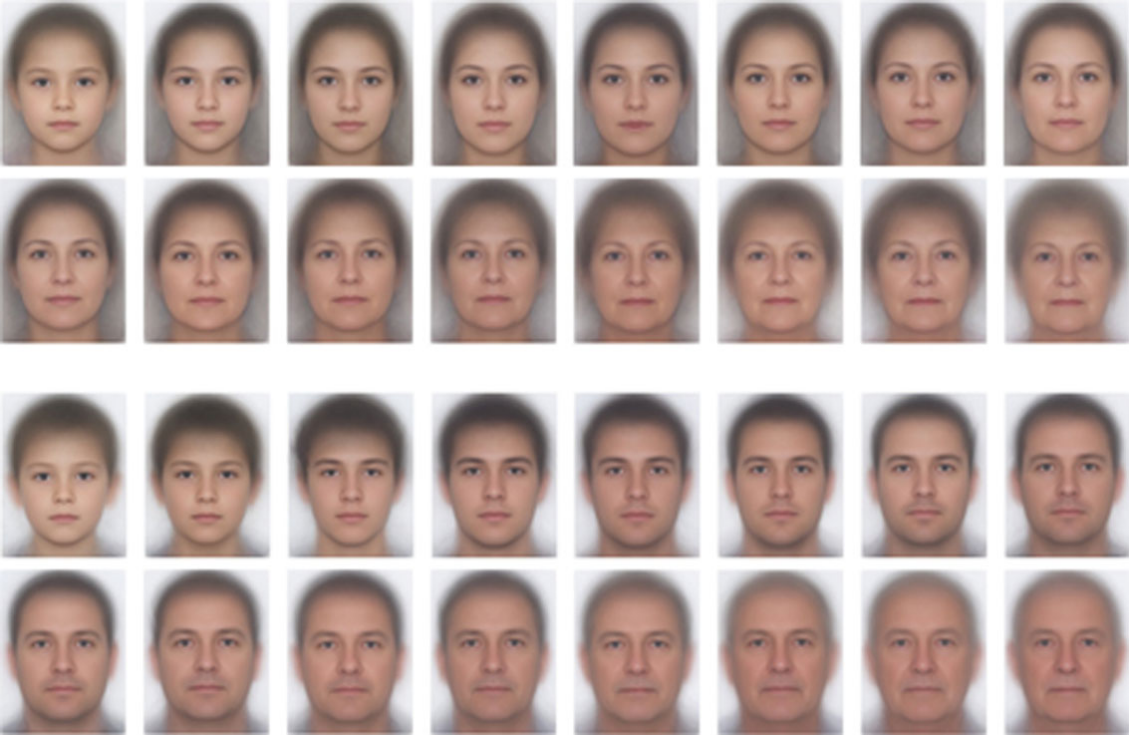
Averages of the individual face images used in the age estimation tasks. Each image is an average of 124 images of a single gender from a range of 4 years. To protect the identity of the people contributing their passport image for use in the study, we were unable to publish individual images that were actually used in the study. The facial averages shown here are for illustration only.

To overcome these limitations, we used a very large database containing passport images of 3968 identities. For each age between 7 and 70, we had images of 62 different faces, meaning that we could ensure that the accuracy of age estimates to that age was not influenced by any particular face, and whether or not it had ‘aged well’. Using passport images also enabled a more naturalistic test of face age estimation, as the demographic make-up of this dataset was reflective of the Australian population, which was the same population from which we sampled study participants. Figure 1 shows average images created using a combination of automatic face detection and image morphing software. Each average in this figure was created using faces from a 4 year age range. Interestingly, this figure gives the subjective impression of the same face getting older, suggesting that by sampling a large pool of faces at each age range we have stabilized the average appearance of the set. We interpret this as evidence that our sampling method was successful in overcoming the limitations outlined above.

## 2. Methods

### 2.1. Participants

Participants in Experiments 1 and 3 were undergraduate psychology students at UNSW Sydney who received course credit for their participation. Participants in Experiment 2 were members of the local community registered with UNSW Sydney's online recruitment system who were paid $15. Across all three experiments, a total of 84 participants were tested, from whom the data of three had to be excluded for non-compliance with task instructions. Participant details are summarized in table 1. As is typical of junior undergraduate students, the age of participants in Experiments 1 and 3 was around 19. To investigate whether participant age had an effect on age perception, participants for Experiment 2 were recruited with a minimum age of 34.

**Table 1:** Participant demographics for each experiment.

Expt #	participants tested	excluded	included (M/F)	age range (mean)
1	30	0	30 (13/17)	18–24 (19.2)
2	20	2	18 (4/14)	34–59 (41.1)
3	34	1	33 (13/20)	18–26 (19.0)

### 2.2. Materials

Stimuli were sampled from a database of 20 000 passport photographs of 20 000 Australian citizens who had consented for their photograph to be used in research during the passport application process. Passport images are valuable as naturalistic stimuli because they are representative of the population of faces, and of the type of reference imagery that age estimation judgements are likely to be performed on in real-world tasks. In addition, image parameters of passport images are relatively standardized and passport images used in this study conformed to international guidelines [25]. High-resolution photographs measuring 35–40 mm in width and 45–50 mm in height were digitally scanned at high-resolution (300 dpi) by the Australian Passport Office. Scanning procedures were compliant with international guidelines of the International Civil Aviation Organization that aim to ensure optimal operation of biometric technology. Resultant digital images measured 426 by 536 pixels.

On the basis of meta-data generated by face recognition software, we excluded 5810 faces that were wearing glasses or had their mouth open. From the remaining set, we randomly sampled 31 males and 31 females at each age in the range 7–70 years, giving a total of 3968 images. Figure 1 shows image averages of experimental stimuli.

**Figure 2.**
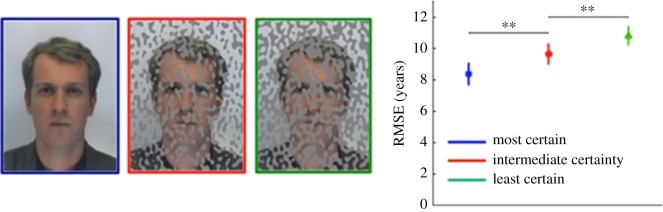
Example of the mask used to manipulate uncertainty (left) and root mean square errors (RMSEs) in facial age estimation as a function of stimulus certainty in Experiment 1 (right), **p < 0.01.

To manipulate uncertainty in the visual stimulus, images were presented at one of three levels of masking: most certain (no mask); intermediate certainty (one-third masked); most uncertain (half masked), illustrated in figure 2. Masked regions of the image were replaced by grey pixels. The masks were generated by thresholding low-pass filtered noise patterns. Specifically, white Gaussian noise was low-pass filtered with a cut-off frequency of three cycles per degree (approx. 18 cycles per face) and then thresholded to form a mask covering the requisite proportion of pixels in the stimulus image. The resulting percept was such that the faces appeared to be behind a partially transparent occluding surface such as glass splattered with mud (figure 2). This means of manipulating stimulus uncertainty was chosen for two principal reasons. Firstly, it did not require any changes to be made to the contrast or texture of the visible regions of the face, properties that are known to bias perceived age [26,27]. Secondly, the visual system naturally tends to complete the occluded regions of the image where local information is unavailable, maintaining the sense that there is a whole face present in the scene and reducing the possibility that a different strategy might be employed to assess the age of the manipulated images.

### 2.3. Procedures

Participants were asked to rate the perceived age of the face using a two-digit integer (between 01 and 99) entered on the number pad of a computer keyboard. Participants were not informed about the age range of the stimuli and no feedback was given as to the accuracy of their responses. On each trial, a single passport image was presented on a 15″ computer monitor with resolution 1920 by 1080, at a size of approximately 7 × 9° of visual angle. Images remained on the screen until the participant had made their response. Each participant completed 384 such trials (64 ages × 2 sexes × 3 levels of stimulus certainty). Stimulus presentation was counterbalanced as far as possible such that no participant saw the same stimulus identity more than once and, across participants, each stimulus identity at each level of uncertainty was presented the same number of times (to within one presentation).

In Experiments 1 and 2, trials were completed in a single block and participants were free to take breaks during the experiment between trials. In Experiment 3, participants completed two blocks of half the length of those in the other experiments. One block in Experiment 3 was composed of stimuli with ages in the range 7–38 and the other 39–70, in order to investigate whether there was any range effect. The order in which the blocks were completed in Experiment 3 was counterbalanced across participants. On average the task took between 30 and 45 min to complete.

### 2.4. Statistical analysis

For statistical analysis, bootstrap resampling with replacement [28] was carried out across participants to create a surrogate dataset of the same size as the original. The process was repeated to create 1000 such datasets. The same analyses were then run on these surrogate datasets as on the original to establish 95% confidence intervals on individual measures and parameter estimates.

Pairwise comparisons between conditions were carried out by comparing bootstrapped difference distributions with the null of zero difference. The number of bootstrapped differences of each sign, *k*_+_ and *k*_−_, were counted. The *p*-value was then estimated as

$$p=\;\frac{\min(k_{+},\;k_{-})+1}{500},$$

such that a bootstrapped difference distribution that did not include zero would be assigned a value of *p* < 0.002.

### 2.5. Computational modelling

In modelling the perception of age from the image of a face, we considered two potential sources of bias captured by a total of four model parameters. The first source of bias was the possibility that participants would have a systematic tendency to over- or underestimate age, and that this tendency might itself vary with the age of the face stimulus [14]. To this end, we modelled perceived age as a linear function of stimulus age, *f*_0_. This linear function was described by two model parameters, the ‘overall bias’, *b*_overall_, in age across the range of stimulus ages (7–70 years) and the ‘slope’, *s*, such that the systematic bias in age, *b*_systematic_, is given by

$$b_{\mathrm{systematic}}(\;f_{0})=b_{\mathrm{overall}}+\;(\;f_{0}-38.5)\;*\;s,$$

where 38.5 years is the mean of the range of stimulus ages. Veridical perception would correspond to an overall bias of zero and a slope of one.

The second source of bias that we modelled was serial dependence, i.e. the tendency for the perception of the current stimulus to be influenced by the immediately preceding stimulus [15–22]. To capture serial dependence in the data, we modelled the (signed) error in perceived age as a derivative of Gaussian function of the difference in age between the previous and the present stimulus. Underlying the use of a derivative of Gaussian function to model serial dependence [15] is the assumption that the strength of any interaction between successive stimuli will decrease monotonically and symmetrically with the difference between those stimuli. This qualitative behavior is conveniently described by a Gaussian function, such that the perceived age of a stimulus is effectively modelled as a weighted sum of the present and previous stimuli where the magnitude of the weight attached to the previous stimulus is a Gaussian function of the difference in their ages. For a given weighting, *w*, of the preceding stimulus, *f*_−1_, the bias it induces, *b*_serial_, on the perceived age of the present stimulus, *f*_0_, will be proportional to the difference in their ages:

$$\begin{array}{rl} b_{\mathrm{serial}}(\;f_{-1}\to \;f_{0}) & =[w*f_{-1}+(1-w)*f_{0}]-f_{0}\\ & =w*(\;f_{-1}-f_{0}). \end{array}$$

Thus, if the weight attached to the previous stimulus is a Gaussian function of the difference in their ages, then the bias it induces will be a derivative of Gaussian function of that difference, i.e. if:

$$w∼\exp\;\left[-\frac{1}{2}{\left(\frac{\;f_{-1}-f_{0}}{\sigma }\right)}^{2}\right]$$

then:

$$b_{\mathrm{serial}}(\;f_{-1}\to \;f_{0})∼(\;f_{-1}-f_{0})\;*\;\exp\;\left[-\frac{1}{2}{\left(\frac{\;f_{-1}-f_{0}}{\sigma }\right)}^{2}\right],$$

where *σ* is the model parameter representing the age difference at which the effect of serial dependence is maximal. The fourth model parameter represents the peak magnitude of this serial dependence. Veridical perception would correspond to a peak magnitude of zero. Together, the effects of these two sources of bias sum such their net effect, *b*_net_, is given by

$$b_{\mathrm{net}}(\;f_{0}|f_{-1})=b_{\mathrm{systematic}}(\;f_{0})+b_{\mathrm{serial}}(\;f_{-1}\to \;f_{0}).$$

Geometrically, the fitted model corresponds to a surface in three dimensions where perceived age is modelled as a function of the age of the present stimulus, *f*_0_, and the age difference, *f*_−1_ − *f*_0_, between the previous and present stimulus. However, for the purposes of visualization, the experimental data will be presented in the Results section collapsed across either (i) the possible range of age differences, *f*_−1_ − *f*_0_, between the previous and present stimulus for each age, *f*_0_, of the present stimulus or (ii) the possible range of stimulus ages, *f*_0_, for each difference in ages, *f*_−1_ − *f*_0_, between the previous and present stimulus.

## 3. Results

We examined age estimation across the entire range of ages (Experiment 1 and 2) and estimation across a restricted range (Experiment 3). In each experiment, participants' task was to provide numerical estimates of age to 384 passport images. Images were presented either in their original format, or with one of two levels of masking, as illustrated in figure 2. By parametrizing visual noise in this way, we aimed to manipulate the uncertainty associated with the perceptual judgement. Based on our previous work, we assume that biases which increase with increased uncertainty reflect the perceptual expectation inherent in the processing of the stimuli.

Absolute error in age estimation was quantified as the root mean square (RMS) deviation of rated age from stimulus age across all stimuli and participants. RMSEs from Experiment 1 are shown in figure 2, showing that for the unmanipulated stimuli (the ‘most certain’ condition), RMSE was 8.36 years (95% CI: [7.70, 9.03]). As a check of the strength of our manipulation of uncertainty, we performed pairwise contrasts between adjacent levels of uncertainty by bootstrap resampling the data over participants. For intermediate certainty, RMSE was significantly greater (*p* < 0.002) at 9.64 years (95% CI: [9.03, 10.27]). For the most uncertain condition, RMSE was significantly greater (*p* < 0.002) than for the intermediate condition at 10.79 years (95% CI: [10.24, 11.37]). The pattern and magnitude of the RMSE was similar in Experiment 2 (see the electronic supplementary material, figure S2).

**Figure 3.**
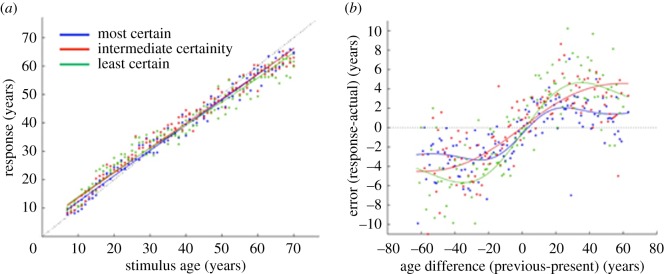
Signed error in Experiment 1. (*a*) Age estimation as a function of stimulus age. (*b*) Signed error as a function of the signed difference in age between the previous and present stimulus. Curves show model fits at each level of stimulus uncertainty.

Having established that our manipulation of uncertainty led to a monotonic increase in the magnitude of error across the three levels, we then looked for systematic patterns of bias (signed error) in data from Experiment 1. Inspection of the data as a function of stimulus age (figure 3*a*) reveals the expected monotonic increase in rated age as a function of stimulus age. However, when the data are compared with the dotted line (denoting veridical responding) in figure 3*a*, it is also evident that there is a tendency for the age of younger faces to be overestimated and for older faces to be underestimated. Furthermore, a plot of the signed error in response as a function of the difference in age between the previous and present stimulus (figure 3*b*) contains data points principally in the lower left and upper right quadrants. This pattern indicates that there is a tendency to underestimate age when the previous face was younger than the present one and to overestimate age when the previous face was older.

To quantify the systematic patterns of bias evident in figure 3, we modelled the data as a combination of two factors: a linear function of stimulus age and a term dependent on the signed difference in age between the previous and present stimulus. The model was able to explain just over 95% of the variance in the data whether fitted to the combined data from all levels of uncertainty or separately to each. Modelling the data in this way allowed us to quantify four distinct features of the data: (A) the overall bias in rated age; (B) the slope of the linear function; (C) the age difference at which dependence on the preceding stimulus is maximal; (D) the peak magnitude of this serial dependence.

For Experiment 1, the best-fitting model parameters at each level of stimulus uncertainty are shown in electronic supplementary material, figure S1. When the data were combined across all levels of uncertainty, overall bias in ratings across the entire range of stimulus ages (7–70) was not significantly different from zero (overall bias: −0.46 years; 95% CI: [−1.29, 0.37]). However, there was a tendency for faces to be rated as older in the intermediate compared with the most certain condition (*p* = 0.014). The slope of the linear function was significantly less than one when fitted to the combined data from all levels of uncertainty (mean: 0.959; 95% CI: [0.885, 0.984]). To give an indication of the magnitude of this effect, it corresponds to a systematic under- (over-) estimation of just over 1.5 years for the oldest (youngest) faces in the range. There were no significant differences between levels of uncertainty for this parameter.

For serial dependency parameters, the age difference at which dependence on the preceding stimulus was maximal was 31.1 years (95% CI: [21.2, 47.1]) for the combined data from all levels of uncertainty. There were no significant differences in this parameter between levels of uncertainty when analysed separately. The peak magnitude of the serial dependence was 3.3 years (95% CI: [2.2, 3.8]) for the combined data from all levels of uncertainty. This corresponds to a significant ‘assimilative’ serial dependence, such that the ratings of age tended to be biased *towards* the age of the preceding stimulus. Pairwise contrasts between adjacent levels of uncertainty revealed that the degree of serial dependence increased with stimulus uncertainty from most certain to intermediate (*p* = 0.018) and from intermediate to most uncertain (*p* = 0.018). Thus, the more uncertain the stimulus, the more its rated age tended to be biased towards the age of the preceding face.

Previous research has shown that the accuracy of face age estimation is modulated by the age of the perceiver [8]. Experiment 1 was conducted on a cohort of undergraduate psychology students with an age range of 18–24, so in Experiment 2 we repeated our initial experiment with a group of older participants (Mean age = 41 years; Range = 34–59). Signed error for this experiment is shown in electronic supplementary material, figure S4 and shows a very similar pattern to the previous experiment, suggesting that age of perceiver did not modulate these effects. When the model was fitted to the combined data from all levels of uncertainty the pattern of results was similar to that for Experiment 1, with no significant differences between fitted parameters across the two experiments (linear slope = 0.970, 95% CI: [0.866, 0.996]; maximal age serial dependence = 34.2 years; peak magnitude serial dependence = 3.9 years). Again, the model was able to explain around 95% of the variance in the data.

**Figure 4.**
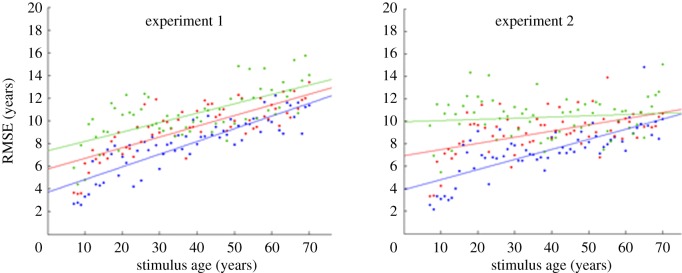
RMSE as a function of stimulus age for participants aged 18–24 (Experiment 1) and 34–59 (Experiment 2), shown with best-fitting straight lines for each level of stimulus uncertainty (blue, most certain; red, intermediate certainty; green, least certain).

The fact that we did not observe any differences in the bias components across Experiments 1 and 2, which involved participants of different age ranges, appears to contradict studies showing an ‘own-age bias’ in age estimation judgements. A number of studies have shown that people are more accurate at estimating ages of faces that are similar to their own age [8,11]; however, these studies report accuracy in absolute error and not signed error. To examine this question more closely, we compared the mean RMSE scores for each of the two age groups tested in Experiments 1 and 2 as a function of stimulus age (figure 4). Participants were generally more accurate with younger faces, but this trend is less marked for the older participant group—consistent with previous studies showing the own-age bias. This pattern was particularly clear for the highest level of stimulus uncertainty (see electronic supplementary material, figure S5).

In Experiment 3, we then tested whether the age range of the *faces* modulated the observed biases. Given that serial dependencies appear to have a substantial impact on age estimation, it is likely that signed errors observed across any given experiment would be dependent on the age range that participants were exposed to. Specifically, we hypothesized a ‘range effect’ such that a given face would tend to look younger when presented within a block of younger faces than when presented within a block of older faces. This hypothesis was based on the observation of an assimilative serial dependence in Experiments 1 and 2, such that judgements of age tended to be biased towards the age of the preceding face. For example, when a middle-aged face is presented within a block of younger faces, the preceding face on any given trial is more likely to be younger and so the net effect of serial dependence across presentations of the middle-aged face should result in a systematic bias for it to look younger.

To test for the range effect predicted on the basis of serial dependence, participants in Experiment 3 completed two blocks of half the length of those in the earlier experiments. One block was composed of stimuli with ages in the range 7–38 and the other 39–70 years. To test whether the range of ages experienced in a given block affected the observed bias, we used linear fits to compare the age bias to 38.5-year-old faces (i.e. the midpoint dividing age ranges in the two blocks; see electronic supplementary material, figure S6). Pairwise comparisons between the fitted responses to blocks differing in stimulus age range revealed significant differences in bias both under the intermediate (*p* < 0.002) and most uncertain (*p* < 0.002) conditions but not the most certain condition. This suggests that serial dependencies cause the perceived age of a face to differ depending on the age range of the cohort being viewed, such that a 38 year old attending an undergraduate lecture will appear younger than the same 38 year old sitting in a professional conference.

## 4. Discussion

Across all three experiments, we show a systematic pattern of bias that accounts for signed errors in estimates of facial age. In general, the age of young faces was overestimated and the age of older faces was underestimated. This result is consistent with previous work showing that age estimation of faces is biased towards the middle-age [14]. Here, however, we have shown that this general bias is mediated by an assimilative serial dependency whereby the perceived age of the current face is biased towards the age of the previous face. Further, the strength of these biases was modulated by the degree of visual noise applied to the stimulus, such that the combined effect was strongest when the percept was most uncertain. Based on this pattern of results, we propose that biases reflect a shift of perception towards stored representations that are shaped by both recent and normative perceptual experience.

An emerging body of recent evidence shows that perception of the face currently viewed is modulated by the faces viewed immediately prior [15–22]. Consistent with these findings, we have shown that the vast majority of perceptual bias can be explained as a shift towards the prior face. Interestingly, depending on the attribute that is being judged, serial dependencies can either serve to make the face appear more similar (assimilative dependencies) or more dissimilar (contrastive dependencies). Assimilative dependencies have been shown for judgements of gender [17], identity [16] and attractiveness [18–22], whereas contrastive effects have been shown for judgements of facial expression [17]. This has led researchers to propose that this intriguing duality arises from adaptive processes that serve to fuse stable properties in the presence of visual noise, through perceptual assimilation, and enhance discrimination between information that varies in time, through contrast effects [17].

Our experiments confirm that perception of age is subject to assimilative serial dependencies. Of course, age is not a stable property, but is one that produces systematic changes in appearance over a relatively long period of time. Therefore, these results suggest that the key to determining whether a property is subject to assimilative or contrastive dependencies is the time course of change. This possibility has been raised previously [17], and our results serve to confirm the plausibility of this account. However, this account, and the assimilative nature of the dependencies we report, stands in contrast to a number of studies showing perceptual adaptation to facial age [25,29,30]. In these studies, adapting to an age range has the *opposite* effect to the serial dependencies reported here—adapting to a young or old face caused faces of an intermediate age to appear older or younger, respectively. This difference is consistent with the notion that weak or brief stimulation (e.g. by the test stimulus on the previous trial) serves to prime perception whereas strong, prolonged stimulation tends to habituate neural mechanisms (e.g. [31]). We did not examine the effects of stimulus duration here but believe this is worthy of systematic study in future.

Importantly, previous accounts of both perceptual adaptation and serial dependencies are based on the assertion that they serve an adaptive function (e.g. [17,32]). However, on the task we report here, this assimilative bias does not appear to be adaptive at all. Rather, when asking participants to make absolute age judgements, serial dependencies were shown to cause non-veridical responses. Given that absolute judgements of age are critical in a number of legal processes—for example, to uphold age restriction laws, when establishing the age of criminal suspects or verifying whether an individual is a minor—these errors can have serious consequences. Using a large set of naturalistic face images, we found a relatively high degree of error, with responses deviating from veridical by an average of 8 years. This is larger than previous estimates of error in this task that have used smaller sets of stimuli captured in laboratory conditions. Having identified systematic biases giving rise to these errors, this new understanding should provide the basis for improving accuracy of age judgements in applied contexts. For example, future research might profitably examine the effect of intervening masking stimuli in moderating biases due to serial dependency.

## Supplementary Material

Supplementary Information

## Data accessibility

Data available from the Dryad Digital Repository: https://doi.org/10.5061/dryad.82fm72s [33]. To protect the identity of the people contributing their passport image for use in the study, we are unable to make available individual stimulus images.
