# Supplementary material for: Two sources of bias explain errors in facial age estimation
Source: R Soc Open Sci. 2018 Oct 17;5(10):180841. doi: 10.1098/rsos.180841 (PMC6227935; doi:10.1098/rsos.180841)
Supplement: Supplementary Information [file rsos180841supp1.docx]

*Supplementary Information:*

**Two sources of bias explain errors in facial age estimation**

Colin Clifford^1^, Tamara Watson^2^ & David White^1^

^1^ UNSW Sydney

^2^ Western Sydney University

**Supplementary results: Experiment 1**


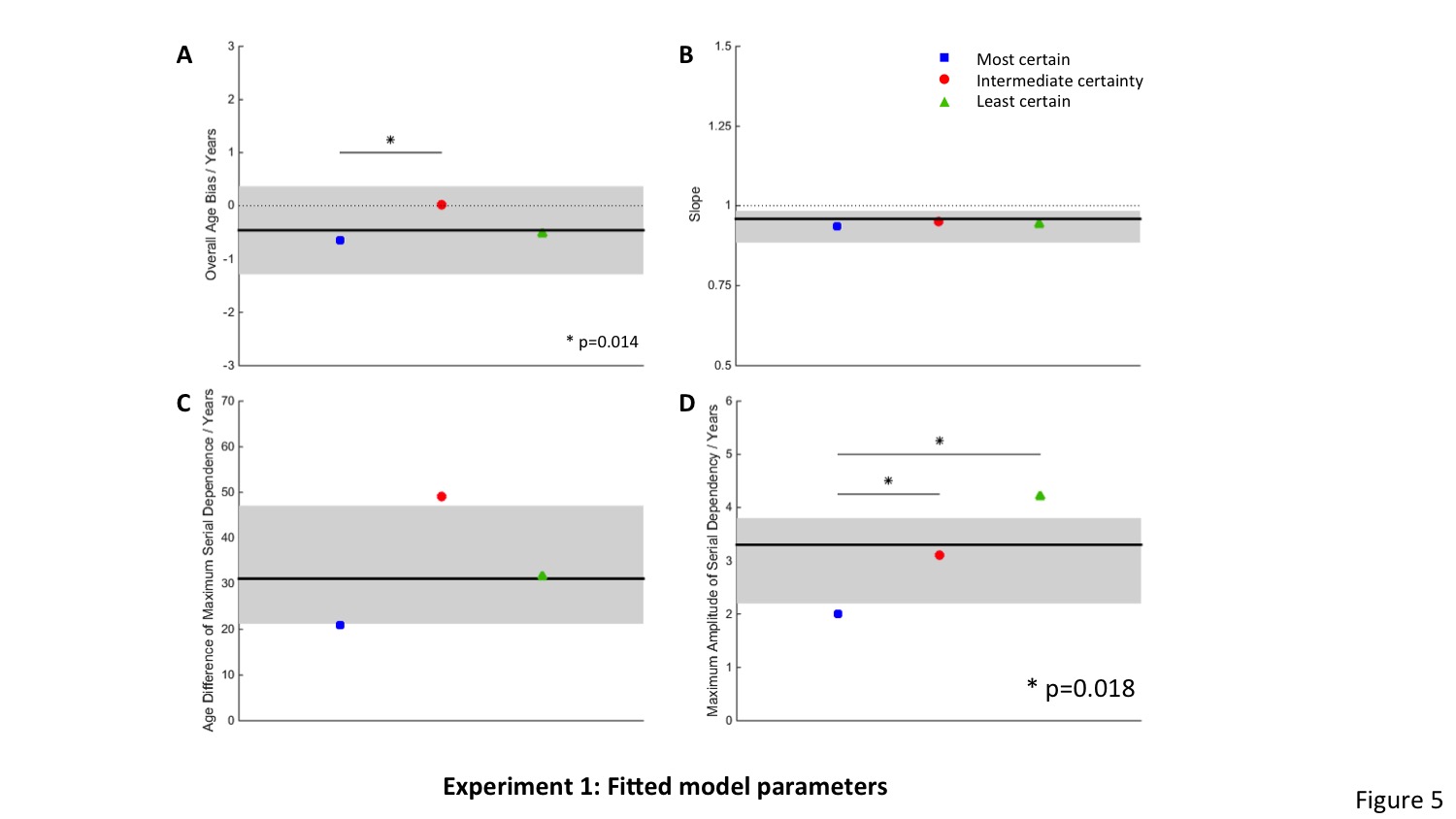


*Supplementary Figure 1. Fitted model parameters from Experiment 1: (A) overall age bias; (B) slope of linear component; (C) age difference of maximum serial dependency; (D) maximum amplitude of serial dependency. In each panel, black line shows best fit to data combined across levels of uncertainty and grey region indicates 95% confidence intervals in that fit. Coloured symbols represent values of parameters fitted to data from individual levels of uncertainty, as per legend top right.*

Further analyses revealed no significant difference in the best-fitting model parameters when the data were broken down by the degree of uncertainty in the *preceding* rather than the present stimulus, nor when broken down by the sex of the stimulus.

**Supplementary results: Experiment 2**

The previous experiment was conducted on a cohort of undergraduate psychology students with an age range of 18-24. To investigate whether the observed effects generalized to older participants, those in Experiment 2 had an age range of 34-59.


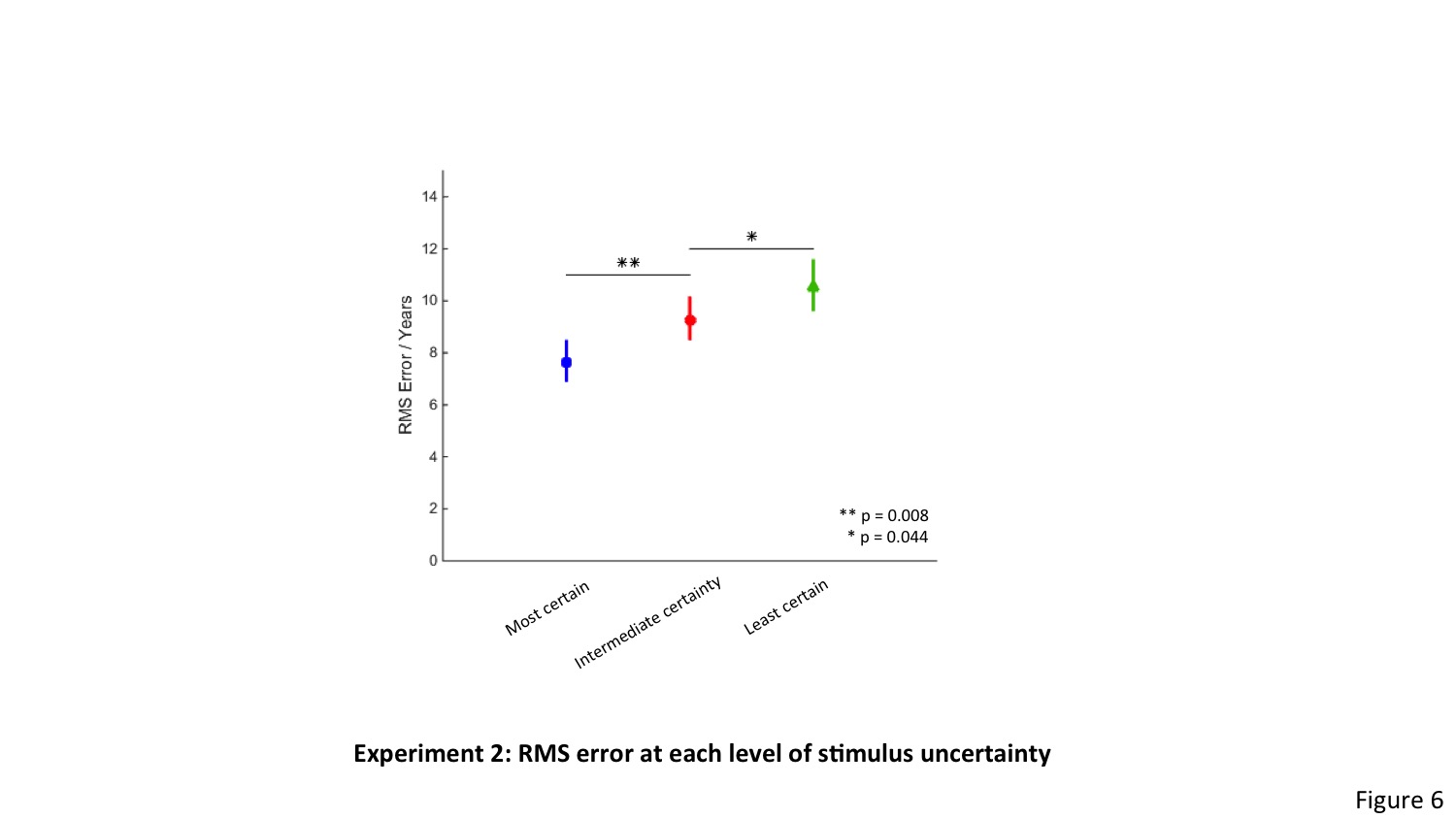


*Supplementary Figure 2. Root mean square errors in facial age estimation as a function of stimulus certainty in Experiment 2.*

Supplementary Figure 2 shows the RMS error for each level of stimulus certainty. RMS error for the most certain condition was 7.65 years (95% CI: [6.94, 8.44]). For intermediate certainty, RMS error was significantly greater (*p* = 0.008) at 9.25 years (95% CI: [8.55, 10.11]). For the most uncertain condition, RMS error was significantly greater (*p* = 0.044) than for the intermediate condition at 10.52 years (95% CI: [9.65, 11.54]). Thus, as in Experiment 1, our manipulation of uncertainty led to a monotonic increase in the magnitude of error across the three levels.


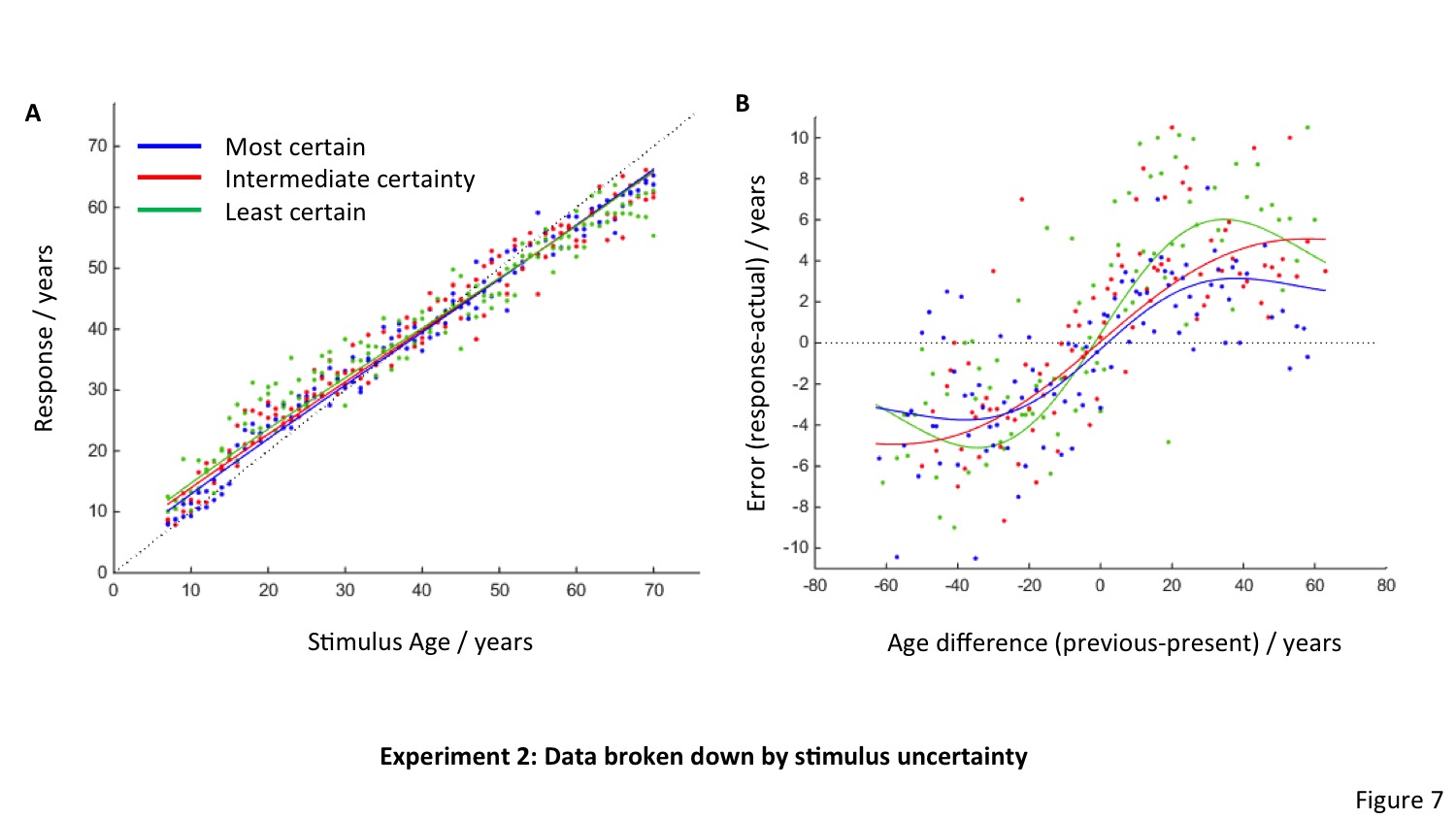


*Supplementary Figure 3. Signed error in Experiment 2. A: Age estimation as a function of stimulus age. B: Signed error as a function of the signed difference in age between the previous and present stimulus*. *Curves show model fits at each level of stimulus uncertainty.*

Inspection of the data as a function of stimulus age (Supplementary Figure 3A) reveals qualitatively the same trends as for Experiment 1: a monotonic increase in rated age as a function of stimulus age with a tendency for the age of younger faces to be overestimated and for older faces to be underestimated. Similarly, the data on serial dependence (Supplementary Figure 3B) follow the trend observed in Experiment 1 to underestimate age when the previous face was younger than the present one and to overestimate age when the previous face was older.


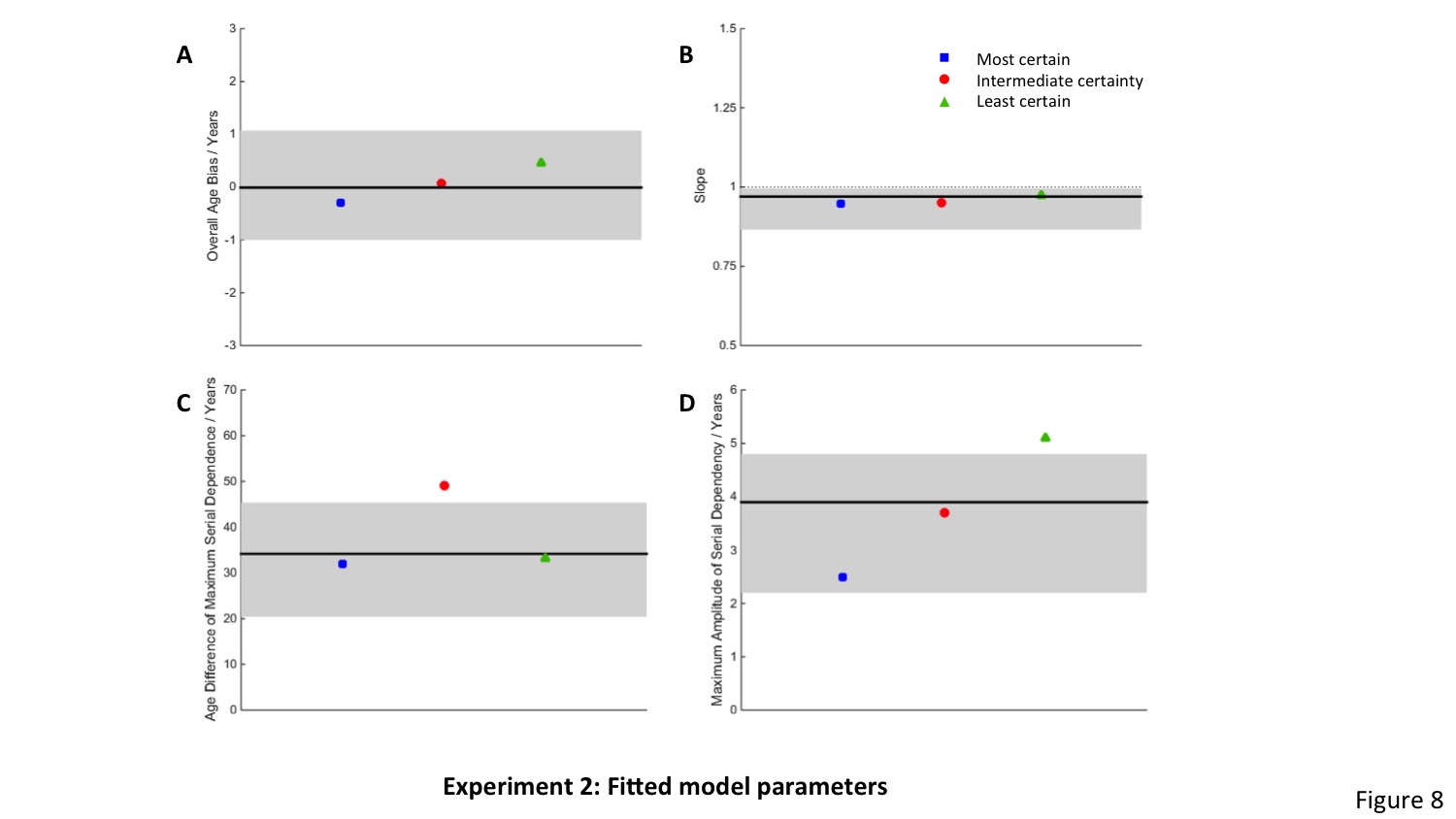


*Supplementary Figure 4. Fitted model parameters from Experiment 2: (A) overall age bias; (B) slope of linear component; (C) age difference of maximum serial dependency; (D) maximum amplitude of serial dependency. In each panel, black line shows best fit to data combined across levels of uncertainty and grey region indicates 95% confidence intervals in that fit. Coloured symbols represent values of parameters fitted to data from individual levels of uncertainty, as per legend top right.*

As shown in Supplementary Figure 4A, there was again no overall bias in rated age (mean: -0.01 years; 95% CI: [-1.00, 1.07]). The slope of the linear function (Supplementary Figure 4B) was again significantly less than one when fitted to the combined data from all levels of uncertainty (mean: 0.970; 95% CI: [0.866, 0.996]). The age difference at which dependence on the preceding stimulus was maximal was 34.2 years (95% CI: [20.4, 45.4]), as shown in Supplementary Figure 4C. The serial dependence was again assimilative (Supplementary Figure 4D), with a maximum amplitude of 3.9 years (95% CI: [2.2, 4.8]).

Unlike in Experiment 1, there were no significant differences between levels of uncertainty for any of the parameters. However, the same qualitative trends were evident. Specifically, there was again a tendency for faces to be rated as older in the intermediate compared to the most certain condition and, again, the more uncertain the stimulus, the more its rated age tended to be biased towards the age of the preceding face.


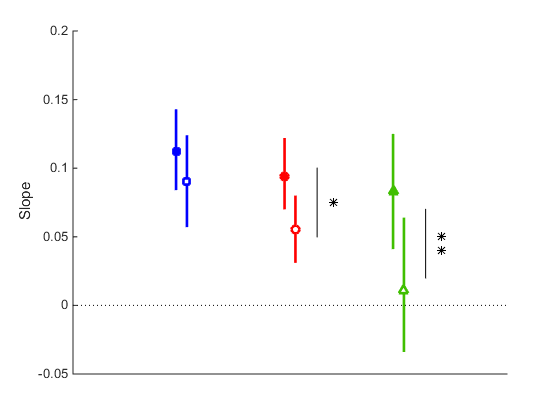


Filled symbols – Experiment 1

Open symbols – Experiment 2

* p = 0.028

** p = 0.024

Most certain

Intermediate certainty

Least certain

*Supplementary Figure 5. Slope of linear fits to RMS Error as a function of stimulus age for participants aged 18-24 (Experiment 1) and 34-59 (Experiment 2). The rate of increase in error with stimulus age is greater for the younger participants, indicating a tendency towards greater accuracy with own-age faces.*

**Supplementary results: Experiment 3**

Experiments 1 and 2 revealed no significant effect of the age range of participants on judgments of facial age. In Experiment 3 we tested whether the age range of the stimulus faces had an effect. Given that a range effect was hypothesized on the basis of modelling of the data from Experiments 1 and 2, we chose a method that was independent of the model to evaluate the statistical significance of the effect. We performed separate linear fits to the data from each block at each level of stimulus uncertainty and then compared the values at which these linear fits intercepted the division line between blocks. In this way we obtained objective estimates of the bias in age ratings at 38.5 years of age.


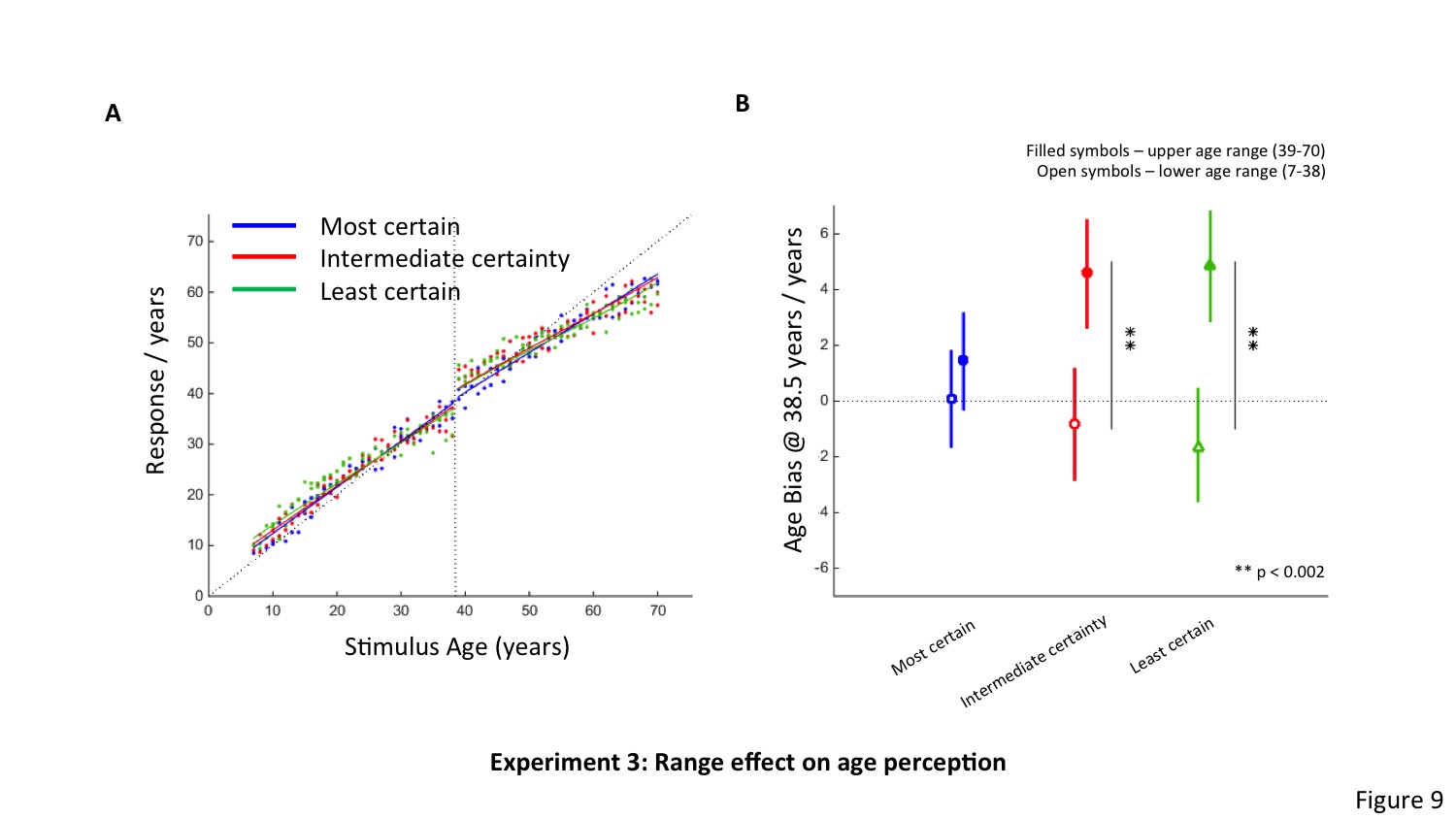


*Supplementary Figure 6. Signed error in Experiment 3. A: Age estimation as a function of stimulus age. B: Bias in age ratings at 38.5 years of stimulus age as a function of stimulus uncertainty and block.*

The data are shown as a function of stimulus age in Supplementary Figure 5A, with the division between the two blocks indicated by the vertical dotted line (at 38.5 years of age). Inspection of the data from the intermediate certainty and least certain conditions in the vicinity of the division between blocks reveals that in the age range just to the left of the division (<=38), faces tend to be rated as younger than their actual age, whereas in the age range just to the right of the division (>=39) they tend to be rated as older. This trend is less evident for the most certain condition.
